# VEGF-C improves regeneration and lymphatic reconnection of transplanted autologous lymph node fragments: An animal model for secondary lymphedema treatment

**DOI:** 10.1002/iid3.32

**Published:** 2014-11-17

**Authors:** Lia Schindewolffs, Gerhard Breves, Manuela Buettner, Catarina Hadamitzky, Reinhard Pabst

**Affiliations:** 1 Institute of Immunomorphology, Hannover Medical SchoolHannover, Niedersachsen, Germany; 2 Department of Physiology, University of Veterinary Medicine, Foundation HannoverHannover, Niedersachsen, Germany; 3 Institute of Functional and Applied Anatomy, Hannover Medical SchoolHannover, Niedersachsen, Germany; 4 Clinic of Plastic, Hand and Reconstructive Surgery, Hannover Medical SchoolHannover, Niedersachsen, Germany

**Keywords:** autologous transplantation, lymph nodes, rat, secondary lymphedema, VEGF-C

## Abstract

Secondary lymphedema occurs after for example breast cancer surgery and radiation in 20–50% of the patients. Due to the poor outcomes of surgical treatments in the past, the therapy often remains symptomatic. However, avascular transplantation of autologous lymph node fragments (LN-Tx) combined with postoperative injections of vascular endothelial growth factor-C (VEGF-C) emerges as a potential surgical therapy. In this study, adult rats underwent LN-Tx to investigate the following parameters of VEGF-C application: time point, location and dosage. Furthermore, the influences of VEGF-C on lymphatic reconnection and transplant regeneration were analyzed. The reconnection was investigated using intradermally injected blue dye and the regeneration was evaluated histologically using hematoxylin-eosin (H&E) staining and immunohistochemistry. The higher dosage enhanced the reconnection rates significantly and showed a statistical tendency of improving regeneration. An application on early postoperative days and the injection into the medial thigh improved the reconnection significantly. However, these variables did not affect the regeneration statistically. This study confirms that LN-Tx combined with lymphatic growth factor VEGF-C is a possible approach in the therapy of secondary lymphedema and shows the important role of VEGF-C application parameters.

## Introduction

The lymphatic system plays an important role in the human body. On the one hand, it is responsible for large parts of the immune defense. Invader antigens are phagocytized by macrophages for direct elimination and also picked up by dendritic cells (DC) for antigen presentation. These antigen-loaded DC are transported into regional lymph nodes (LN) through the lymphatic system. As secondary lymphoid organs, LN host immune cells such as the cortically located B-lymphocytes and the paracortically located T-lymphocytes. Therefore, activation of lymphocytes through DC also takes place in the LN. Due to the cell trafficking through the LN, the histological architecture is essential for its function. Recently, the histotopographic localization of immune cells as well as lymphatic endothelial cells and its relevance for the cellular transport within the LN have been described [1,2] and excellently reviewed [3].

On the other hand, the lymphatic vessels collect accumulated interstitial fluid and are therefore responsible for the regulation of tissue fluid homeostasis [4]. Exact data on lymph flow in individual tissues is not available from studies in humans or animals. This is mainly due to problems of methodology and varied techniques of quantitative lymph collection [5]. The overall lymph flow in humans has been estimated to range between 2 L [6,7] and up to 8 L [8] per day. Some of the fluid is reabsorbed by initial lymphatics. However, since the lymph osmolarity is lower in the afferent lymphatics than in the efferent [9], most of it seems to be returned to the blood system within the LN. Ohtani et al. [9] assumed that high endothelial venules (HEV) are responsible for this fluid resorption.

Considering the amount of accumulating fluid, the need for a functional lymphatic system, and especially functional LN, is obvious. Disturbances can lead to an imbalance of fluid homeostasis and therefore cause accumulation of lymph in the extracellular space: lymphedema, a condition in which the quality of life is greatly restricted and patients experience pain, immobility and poor body image [10]. Primary causes of lymphedema are genetic disorders such as Milroy Disease, which is caused by a mutation of the VEGF-receptor-3 (VEGFR3) and results in a hypoplasia of the lymphatics and a primary lymphedema [11,12]. Secondary causes are more prevalent and can be the consequence of bacterial or parasitic (filarial) infections [13] or the surgical removal of LN and lymphatics, for example, during axillary LN dissection (ALND) due to breast cancer. Since sentinel lymph node biopsy (SLNB) developed as the method of choice for the axillary staging of women with early breast cancer [14], fewer patients require ALND. However, ALND is still indicated when metastases are present. After ALND, up to 30% of the patients develop secondary lymphedema [15]. After SLNB up to 22% of patients still suffer from arm swelling and lymphedema [14], consequently incidence rates remain high, as very recently pointed out [16]. Despite invasive surgical interventions, several risk factors, especially genetic dispositions [17–21], a high body-mass index and low physical activity, additionally increase the occurrence of secondary lymphedema [22].

Most of the surgical techniques, such as lymphatic bypasses and flap interpositions did not show convincing long-term effects [23–25]. Vignes et al. [26] recently stressed the high rate of complications when autologous lymph nodes with surrounding fat tissue were transplanted, although it was performed in a specialized medical center. An exception is liposuction in late-stage lymphedema, resulting in long-term positive effects when performed in a specialized clinic [27].

Thus, secondary lymphedema therapy often remains symptomatic. However, the transplantation of autologous LN (LN-Tx), which has already been investigated in pigs [28–30] mice [31], sheep [32] and rats [33], appears to be a potential surgical therapy and additional applications of VEGF-C revealed beneficial effects [31,33]. Recently, Sommer et al. [34] developed an adequate animal model for lymphedema and explored the effects of VEGF-C with promising results regarding the reconnection and regeneration of LN [33]. However, the question whether the results (reconnection of transplanted LN with the lymphatic system and histological regeneration of transplanted LN) can be influenced by the application parameters remained. Therefore, our study was based on the animal model used by Sommer et al. [34] and we changed the following application parameters: time point, location and dosage. Further details have been described [35]. The aim was to analyze the influences of these parameters on the reconnection of the transplants with the lymphatic system as well as the histological regeneration of the LN architecture. Adequately integrated LN with a natural histological structure are required for normal lymph drainage and the groundwork for further research in the field of LN-Tx. The results of our study are impressively demonstrating the relevance of application parameter modification and provide essential data for following LN-Tx studies.

## Materials and Methods

### Experimental animals

**Figure 1:**
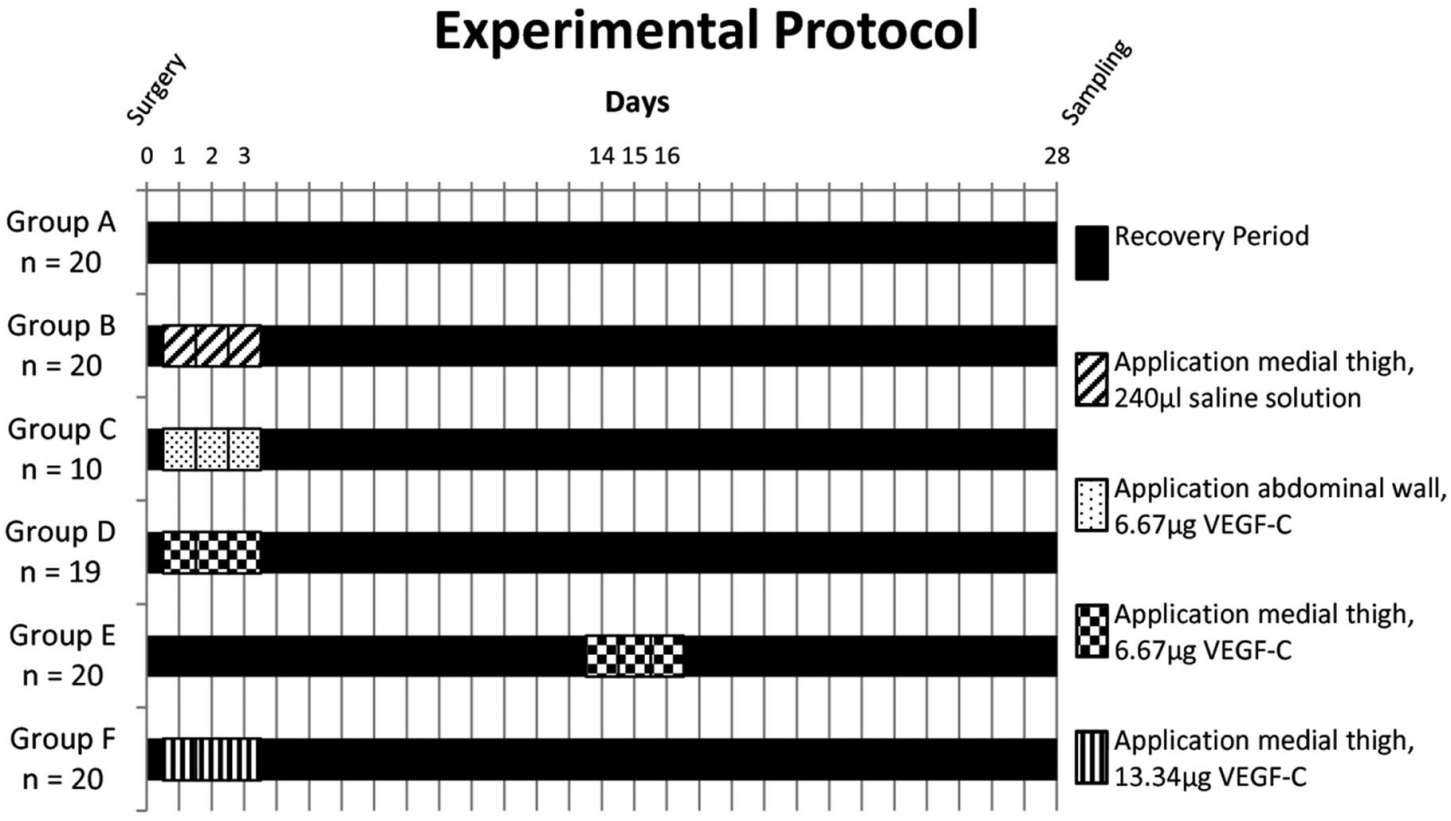
Experimental protocol. In every group one VEGF-C application parameter, such as time point, location or dosage was modified. Group A was left without further intervention after surgery (control group) and group B was treated with 240 µL saline solution (saline control group). VEGF-C, vascular endothelial growth factor-C.

The experimental procedure had been approved by the local authorities (no. 33.9-42502-04-07/1420). For this study, 109 adult female Lewis rats (LEW/Crl, approximately 200 g) were used. The rats were divided into six groups (A–F). Groups A, B, E, and F included 20 rats, group C 10 rats, and group D 19 rats (Fig. 1).

The animals were housed in groups of 5 under specific pathogen free conditions with a 14 h light and 10 h darkness rhythm and were offered total pathogen free nutrition and osmosis filtered water ad libitum.

### Transplantation procedure

All rats underwent isoflurane anesthesia (Isofluran Baxter, Baxter Deutschland GmbH, Unterschleißheim, Germany) after one week of acclimatization and an analgesia injection of 5 mg/kg Carprofen subcutaneously (Rimadyl®, Pfizer GmbH, Karlsruhe, Germany) 30 min prior to surgery. In order to simulate the lesions occurring during breast cancer related lymph node dissection, all draining lymph nodes of one limb were eliminated. Accordingly, all popliteal and inguinal LN on the right side were removed. Three inguinal LN were harvested and cut-off one pole each in order to fragment the transplant. Afterwards, non-absorbable suture (Prolene, 5-0, Ethicon GmbH, Norderstedt, Germany) was used to fix them to the subcutaneous tissue. Therefore, the subcutaneous fat tissue was punctured, all three transplants were stringed to the suture and the tissue was punctured again, thus the suture knots and the transplants were separated by subcutaneous fat tissue. The inguinal and popliteal incisions were closed subcutaneously and with skin stitches using absorbable suture (Marlin® violett 5/0, Catgut GmbH, Markneukirchen, Germany). The animals received 5 mg/kg Carprofen subcutaneously 3 days following surgery.

### Groups

Group A (*n* = 20) functioned as a control group and was left without further intervention after surgery (Fig. 1). The rats in the saline control group (group B, *n* = 20) were given 240 µL saline solution on postoperative days 1, 2, 3 intradermally into the medial side of the thigh (Fig. 1). Groups C (*n* = 10) and D (*n* = 19) were treated with 6.67 µg VEGF-C (VEGF-C rr, ReliaTech GmbH, Wolfenbüttel, Germany) on postoperative days 1, 2, 3 but whereas group C was injected intradermally into the abdominal wall, group D was injected intradermally into the right medial thigh (Fig. 1).

In Group E (*n* = 20) each rat received an injection of 6.67 µg VEGF-C intradermally into the right medial side of the thigh on postoperative days 14, 15, 16 and group F (*n* = 20) was treated as Group D but with an injection of 13.34 µg VEGF-C per rat on postoperative days 1, 2, 3 (Fig. 1). In this group one sample was not evaluated immunohistochemically for technical reasons. All groups were evaluated by the same person (LS) in a blind study.

### Sampling

**Figure 2:**
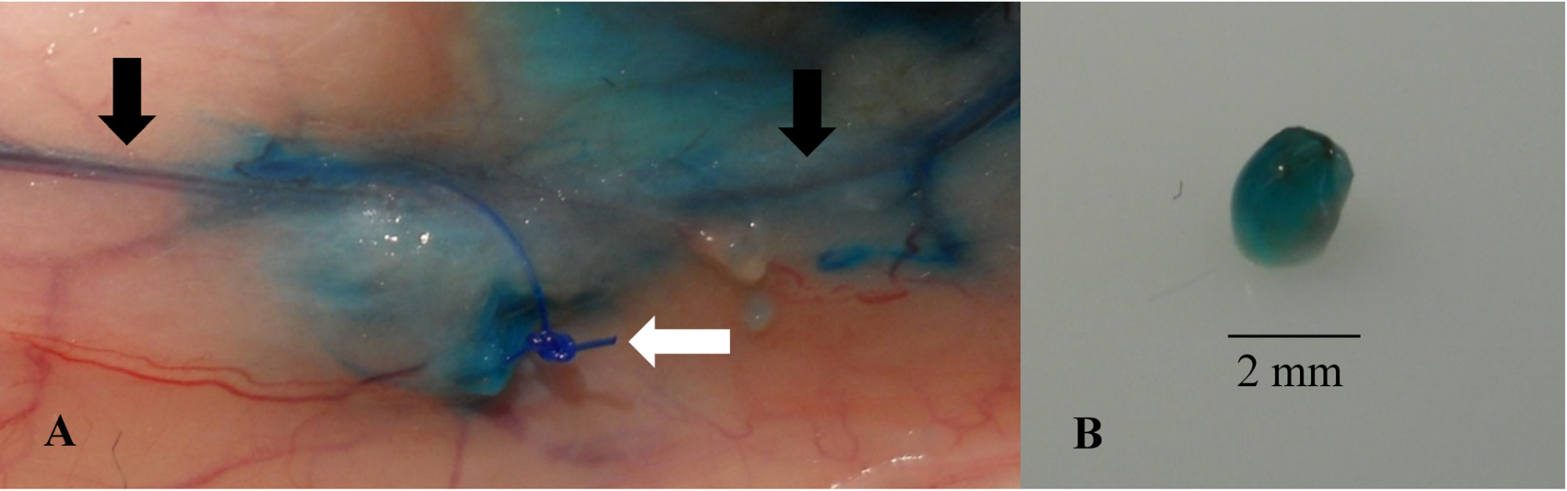
Lymphatic reconnection. (a) Blue stained afferent and efferent lymphatics (black arrows). The transplant, marked with non-absorbable suture (white arrow), is hidden under fat tissue. (b) Stained transplant after removal.

After a period of 4 weeks, the rats were anesthetized with 90 mg/kg ketamine (Ketamin Gräub, Albrecht GmbH, Aulendorf, Germany) and 10 mg/kg xylazine (Rompun®, Bayer Health Care, Leverkusen, Germany) intraperitoneally. A small amount of blue dye (100–300 µL) (Patentblau V, Gerbet GmbH, Sulzbach, Germany) was injected intradermally into the medial right thigh. In order to stimulate the lymphatic flow, extension, and flexion movements as well as gentle massages of the right hind leg were performed for 1–2 min. The rats were sacrificed by cervical dislocation. A major skin cut was made and the skin was spaciously detached to obtain an adequate overview of the distribution and staining of the lymphatics and transplants. The reconnection was evaluated as successful when blue stained afferent and efferent lymphatics were leading to an equally stained transplant (Fig. 2).

### Histochemistry

After removal, the specimens were frozen in liquid nitrogen and 5 µm slices were made of each sample. H&E staining was used for an overview of the LN structure and distribution of lymphocytes. For proper cell identification and hence in order to specify whether a transplant was histologically regenerated, we additionally performed immunofluorescent staining. Therefore, primary and secondary antibodies were used to detect T-lymphocytes, B-lymphocytes, HEV, and lymphatic endothelial cells (Table1). The classification in regenerated LN and not regenerated LN was made according to the existence of the aforementioned lymphocytes (B- and T-cells) and endothelial structures (HEV) as well as the physiological distribution of these cells forming the architecture of the LN.

**Table 1:** Primary and secondary antibodies used

Detected cell	Primary antibodies	Company	Species	Monoclonal
B-lymphocytes	BM 4013	Acris antibodies, Herford, Germany	Mouse	×
T-lymphocytes	R73 FITC (inkcl. sec. AB)	AbD serotec, Düsseldorf, Germany	Mouse	×
Lymphatic endothelial cells	LYVE-1	Relia Tech GmbH, Wolfenbüttel, Germany	Rabbit	×
HEV	HIS 52	Abcam, Cambrige, USA	Mouse	×
	Secondary antibodies			Polyclonal
Lymphatic endothelial cells	CY™3	Dianova GmbH, Hamburg, Germany	Goat anti rabbit	×
HEV	AF 546	Invitrogen GmbH, Darmstadt, Germany	Goat anti mouse	×

### Statistics

For statistical evaluation, *GraphPad Prism* (5.02, GraphPad Software, La Jolla, CA, USA) was used. The groups were combined in different ways (Table2) to analyze the parameters time point, location and dosage with the Fisher's Exact Test. Thus, *P*-values of 0.05 < *P* < 0.10 showed a statistical tendency, a result with *P*-values of <0.05 was significant, with *P* < 0.01 highly significant and with *P* < 0.001 extremely significant.

**Table 2:** Group constellations for statistical purposes

Investigated parameter	Statistically combined groups	Time point	Dosage	Location
Time point	A	No intervention after LN-Tx		
	B	Postoperative days 1, 2, 3	240 μL saline solution lC	Medial thigh
	D	Postoperative days 1, 2, 3	6.67 μg VEGF-ClC	Medial thigh
	E	Postoperative days 14, 15, 16	6.67 μg VEGF-ClC	Medial thigh
Location	A	No intervention after LN-Tx		
	B	Postoperative days 1, 2, 3	240 μL saline solution lC	Medial thigh
	C	Postoperative days 1, 2, 3	6.67 μL VEGF-ClC	Abdominal wall
	D	Postoperative days 1, 2, 3	6.67 μg VEGF-ClC	Medial thigh
Dosage	A	No intervention after LN-Tx		
	B	Postoperative days 1, 2, 3	240 μL saline solution lC	Medial thigh
	D	Postoperative days 1, 2, 3	6.67 μg VEGF-ClC	Medial thigh
	F	Postoperative days 1, 2, 3	13.34 μg VEGF-ClC	Medial thigh

## Results

### Reconnection of the transplants with lymphatic collectors

The injection of blue dye allowed the examination of the integrity of the previously damaged lymphatic system in the upper hind leg and inguinal region (Fig. 2). Only reconnected transplants are able to receive intradermally injected blue dye via the reconnected lymphatic vessels. Transplants which were not reconnected were not stained at all.

In the control group (group A) 15% and in the saline control group (group B) 10% of the rats showed a reconnection of the transplants with the lymphatics. Group C (early application, 6.67 µg VEGF-C, abdominal wall) presented a rate of 50% reconnected transplants and group D (early application, 6.67 µg VEGF-C, medial thigh) a rate of 53%. In group E (late application, 6.67 µg VEGF-C, medial thigh) there were 35% of the rats and in group F (early application of 13.34 µg VEGF-C, medial thigh) 85% presenting reconnected transplants.

**Figure 3:**
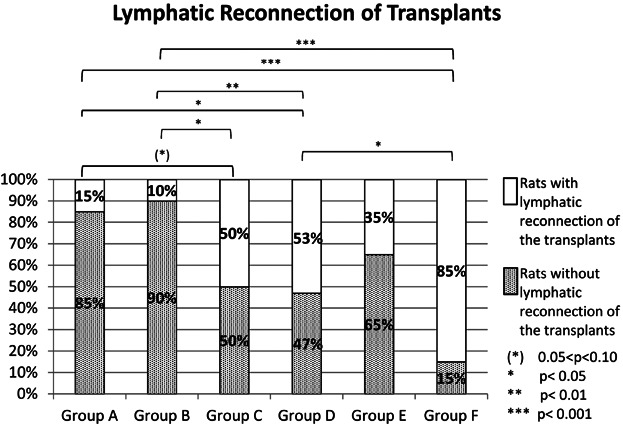
Lymphatic reconnection of transplants and statistical significances.

In terms of the time point, the analysis of group A (control) and group D showed a statistical significance. The comparison between group B and group D was highly significant. The comparison between group A (control) and group E (late application, 6.67 µg VEGF-C, medial thigh) as well as between group D (saline control) and E presented no relevant statistical difference (Fig. 3).

Regarding the location, a statistical tendency was shown between group A (control) and group C (early application, 6.67 µg VEGF-C, abdominal wall) and a statistical significance between group B (saline control) and C as well as between group A (control) and group D (early application, 6.67 µg VEGF-C, medial thigh) (Fig. 3).

The group comparison to investigate dosage influences revealed the following results: Group A (control) and B (saline control) compared to group F (early application, 13.34 µg VEGF-C, medial thigh) showed an extremely significant effect and group A compared to group D presented a statistical significance. The comparison between group D and F also showed a statistical significance (Fig. 3).

### Regeneration of transplants

**Figure 4:**
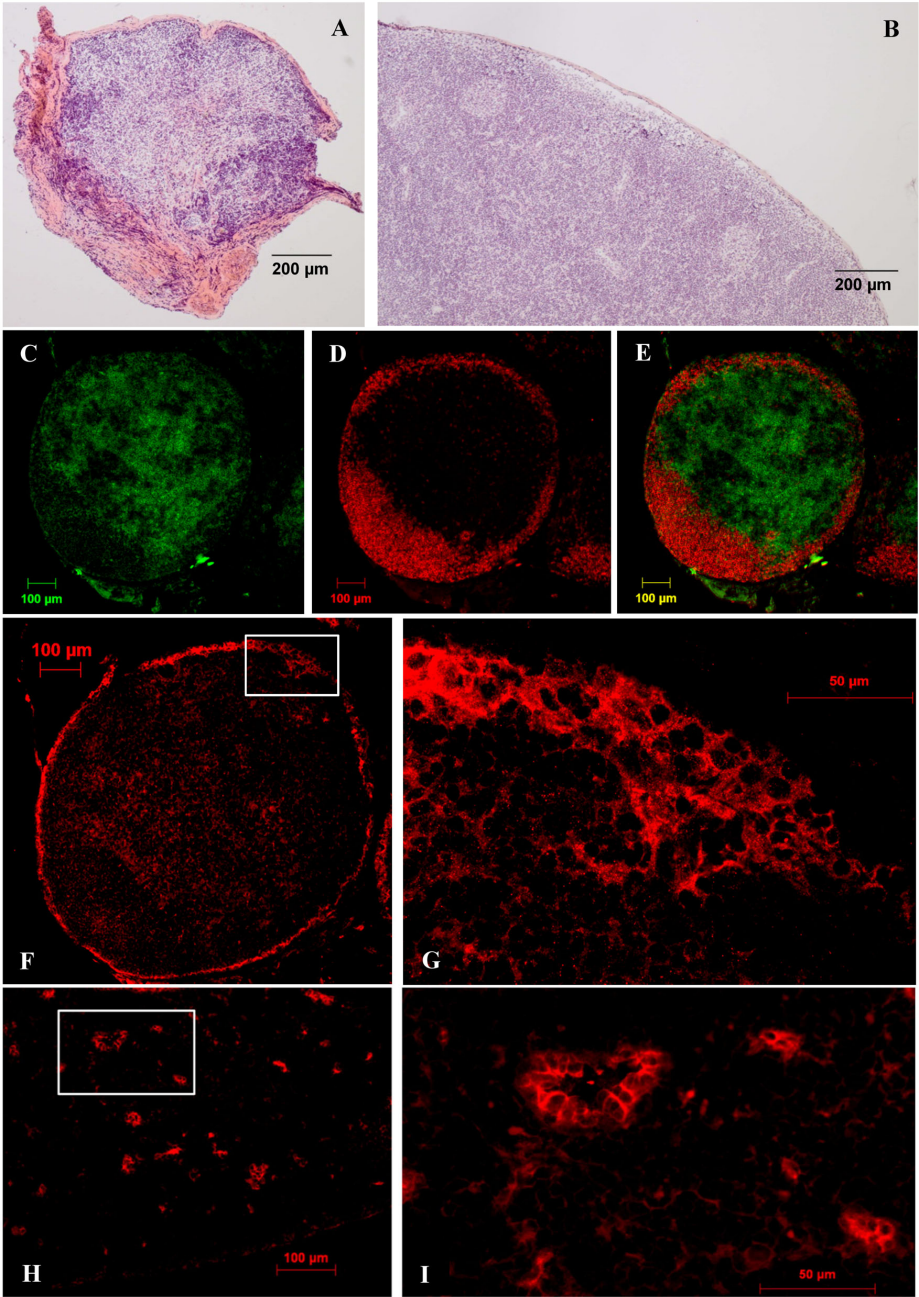
H&E staining and immunohistochemistry. (a and b) H&E staining of a necrotic (a) and a potential regenerated (b) transplant. (c–i) Regenerated transplant after immunohistochemistry. (c–e) Primary antibodies used against T-lymphocytes (R73) and B-lymphocytes (BM 4013) and immunofluorescent secondary antibodies FITC and AF564. (c) T-lymphocytes are located paracortically. (d) B-lymphocytes are located cortically. (e) Merged image. (f and g) Primary antibody used against lymphatic endothelial cells (LYVE-1) and immunofluorescent secondary antibody Cy3. (h and i) Primary antibody used against high endothelial venules (His52) and immunofluorescent secondary antibody AF546. H&E, hematoxylin and eosin.

In order to differentiate between necrotic transplants and potentially regenerated ones, H&E staining was performed (Fig. 4a and b). All of the potentially regenerated transplants had been prepared for immunohistochemistry to examine the distribution of the B- and T-cells (Fig. 4c–e) as well as the presence of lymphatic endothelial cells (Fig. 4f and g) and HEV (Fig. 4h and i) in order to assess whether a transplant was histologically regenerated or not.

In the control group (group A) and in the saline control group (group B) 70% of the rats showed regenerated transplants. Group C (early application, 6.67 µg VEGF-C, abdominal wall) presented a rate of 80% rats with regenerated transplants, group D (early application, 6.67 µg VEGF-C, medial thigh) 89%, group E (late application, 6.67 µg VEGF-C, medial thigh) 85% and group F (early application, 13.34 µg VEGF-C, medial thigh) 95%.

**Figure 5:**
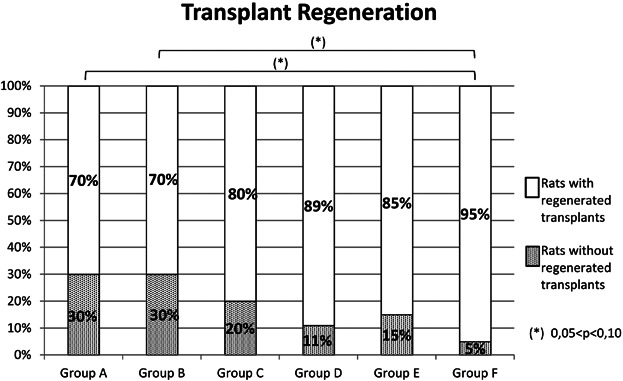
Transplant regeneration and statistical significances.

Regarding the time point, none of the comparisons (control groups A and B, group D and E) showed any statistical relevance (Fig. 5) and the analysis of the parameter location (control group A and B, group C and D) revealed no statistical relevance either (Fig. 5).

In terms of the dosage however, the comparison between group A (control) and group F (early application, 13.34 µg VEGF-C, medial thigh) as well as between group B (saline control) and group F showed a statistical tendency, but the difference between the control groups (A and B) and group D and between group F and D showed no statistical relevance (Fig. 5).

## Discussion

The most common causes for secondary lymphedema in industrialized countries are oncologic surgery and radiation. Prostate [36] and penis cancer in men, cervical, genital and breast cancer [37] in women, but also melanoma of the extremities [38], are examples of high prevalence tumors with a tendency for lymphatic metastasis. To address these malignancies, the removal and/or radiation of axillary/inguinal draining lymph nodes has proved to be an important prognostic factor [39]. Nevertheless, this therapy also deprives the operated areas of their local immune defenses, located in the removed/irradiated lymph nodes. Additionally, 20–50% of these patients develop secondary lymphedema [15,40,41]. LN-Tx of autologous donor lymph nodes, not primarily involved in the tumor drainage area (“clean” nodes), seems to benefit the lymphatic drainage of the receptor area and could potentially be used as a solution for secondary lymphedema. Interestingly, transplanted lymph nodes also have been proven to regain their immune function, including being able to trap tumor cells [for Review see [42]]. Thus, in case of recurrence of the primary malignancy, LN-Tx yields potential benefits in the protection of systemic metastasis by regaining local immune and filtering function, in addition to the potential improvement of lymphedema.

There are also new findings of genetic causes of breast cancer susceptibility [43] and recent studies documented that lymphedema predisposition after breast cancer surgery can be associated with genetic mutations as well [17–21]. Most surgical interventions for lymphedema management deliver no long-term success [23,24], further efforts should be made to understand if LN-Tx could be a potential solution as former publications indicate [28–33,44]. Intradermal application of VEGF-C combined with LN-Tx revealed a beneficial effect on the transplant regeneration and the recovery of the lymphatic integrity [33].

This procedure was tested on several animal model, such as pigs [28–30], mice [31], sheep [32], and rats [33]. Recently, Sommer et al. [34] developed an adequate animal model for lymphedema and explored the effects of VEGF-C application [33]. Their study supported promising results regarding the reconnection and histological regeneration of LN [33].

Our animal model was based on the recently established techniques [33] and the outcomes of our study answer the question on how reconnection of transplanted LN within the lymphatic system and histological regeneration of transplanted LN can be influenced by VEGF-C application parameters.

The reconnection of the transplants to the lymphatic system is the prerequisite to regenerate the possibility for adequate drainage of the limb. The results of this study showed that, regarding the time point, an early application (postoperative days 1, 2, 3) benefits the reconnection significantly in comparison with a late time point (postoperative days 14, 15, 16) (Fig. 3). Although the VEGF-C treatment itself significantly enhanced the reconnection rate, the location did not seem to influence it (Fig. 3). Nevertheless, because of the better *P* values (data not shown) an application into the medial thigh is recommended. Reconnection rates proved to be highly VEGF-C dosage dependent. A high dosage of 13.34 µg enhanced the result highly significantly and also showed a significant advantage over an injection of only 6.67 µg (Fig. 3).

Additionally to the reconnection of the lymphatics, the proper histological regeneration of the LN architecture is essential to create a functional system for efficient lymph drainage. Although the results regarding regeneration of the transplants are less significant than the ones regarding the reconnection, there are some important aspects: time point and location did not present any statistical relevance (Fig. 5), though the better *P*-values (data not shown) also indicate that an early time point and an intradermal application into the medial thigh would be the better option. A high dosage, however, showed a statistical tendency, whereas the lower one did not show any statistically relevant effect. Taken together, in this study an early application on postoperative days 1, 2, 3 of 13.34 µg VEGF-C in the medial thigh improved the reconnection and regeneration of autologous LN transplants. Hence, our work points out the importance of the VEGF-C application parameters when performing LN-Tx. However, to gain more information, the dosage should be tested more intensively. Although in this study no macroscopic changes of the neighbored tissue and/or leakiness of the blood vessels was seen, it is described that this can be a side effect of high VEGF-C dosages [45] and should therefore be investigated in further experiments.

In our study, young and healthy animals were used. As a model for the clinical situation, older animals are recommended for future experiments, because most patients developing secondary lymphedema are over 60 years old [46]. Furthermore, in humans the LN of older individuals show some histological changes. Although the general architecture is still present, generalized degeneration and less demarcated T- and B-zones have been described [47]. We used healthy rats, as we conceived LN-Tx as a possible preventive surgical procedure of lymphedema after breast cancer treatment. This was based on the information that fibrosis is common in chronic lymphedema [10] and therefore the proliferation of lymph endothelial cells is disturbed [48], which means that vasculogenesis is likely not involved in the pathogenesis of lymphedema [49]. Studies to investigate the effects of LN-Tx in chronic lymphedema are required.

Another aspect, which remained unexplored is the lymph drainage efficiency of the animal model over an extended time period of more than 4 weeks. For that purpose, a possible method would be the use of indocyanine green (ICG) in combination with an imaging unit, such as the photodynamic eye built by Hamamatsu Photonics in Japan. ICG is a fluorescent substance which is already used on humans for example for SLN detection [50,51]. Complications with ICG are rare (0.17%) [50] and it can be used repeatedly on one living individual. Serizawa et al. [52] successfully tested this method on a tail model of lymphedema in rats.

Due to the recent developments in research regarding genetic dispositions of most of the secondary lymphedema patients [17–21], we plan the first clinical trial in women with extremely increased likelihood of secondary lymphedema. Our study emphasizes the functionality of VEGF-C associated LN-Tx and confirms that VEGF-C generally has a beneficial impact on the histological regeneration and lymphatic reconnection of the transplants. Moreover, it provides fundamental data for an efficient surgical procedure so that the possibility to establish a long-term surgical treatment for secondary lymphedema becomes more and more concrete.

## Supporting Information

**Figure S1.** Surgical procedure. (A) The subcutaneous tissue was punctured. (B) The three harvested lymph nodes got (C) stringed to the needle and suture and (D) the subcutaneous tissue was punctured again. (E) Due to the surgical technique the transplants lay on a subcutaneous tissue bridge and therefore (F) the knot is separated from the transplants by subcutaneous tissue. **Figure S2.** Transplantation area during sampling.
