# Supplementary material for: VEGF-C improves regeneration and lymphatic reconnection of transplanted autologous lymph node fragments: An animal model for secondary lymphedema treatment
Source: Immun Inflamm Dis. 2014 Nov 17;2(3):152–61. doi: 10.1002/iid3.32 (PMC4257760; doi:10.1002/iid3.32)
Supplement: Supplementary file 1 — Figure S1. Surgical procedure. (A) The subcutaneous tissue was punctured. (B) The three harvested lymph nodes got (C) stringed to the needle and suture and (D) the subcutaneous tissue was punctured again. (E) Due to the surgical technique the transplants lay on a subcutaneous tissue bridge and therefore (F) the knot is separated from the transplants by subcutaneous tissue. Figure S2. Transplantation area during sampling. [file iid30002-0152-sd1.docx]

**Supplementary Data**

Fig. 1.:


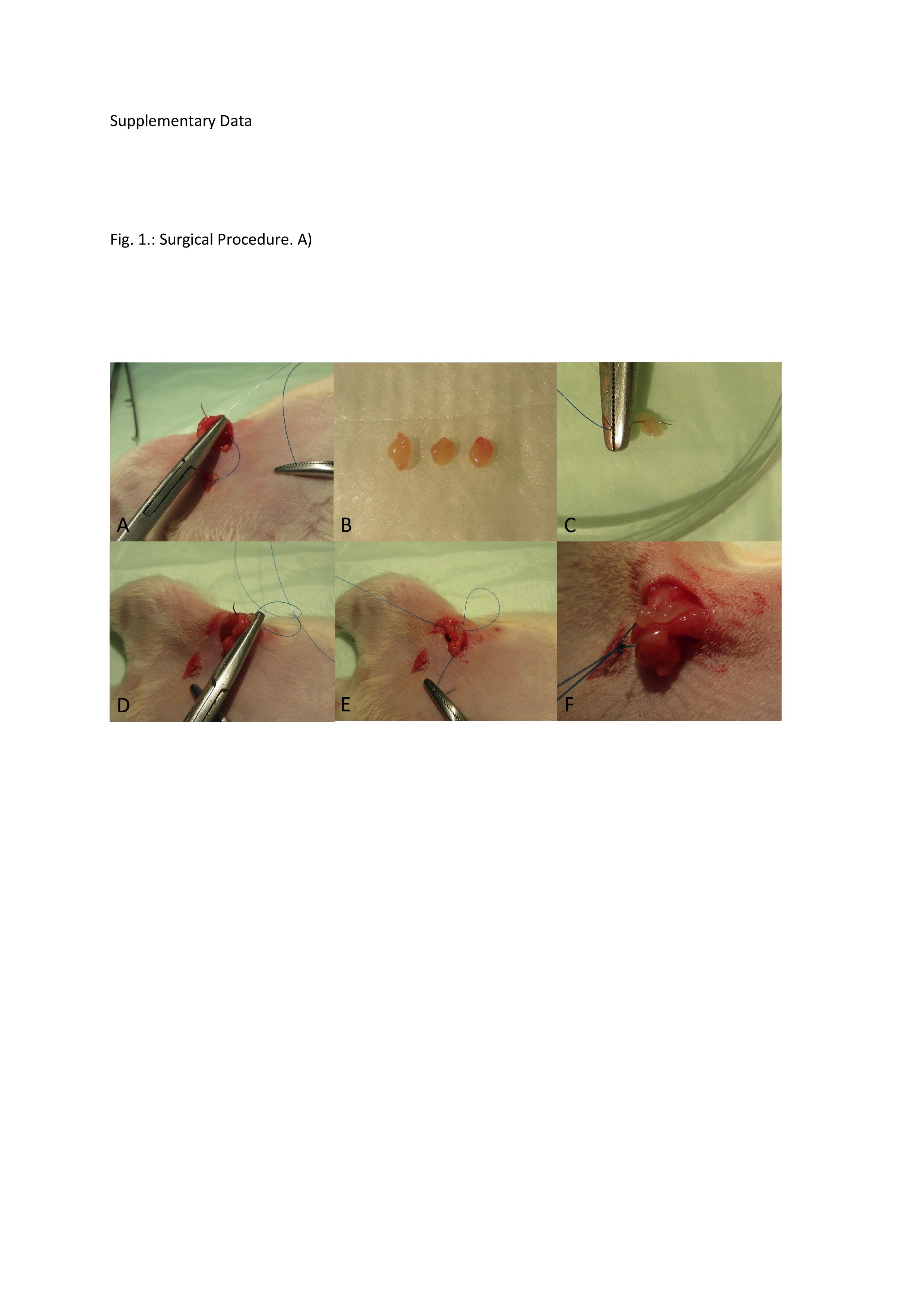


Fig. 1.: Surgical procedure. A) The subcutaneous tissue was punctured. B) The three harvested lymph nodes got C) stringed to the needle and suture and D) the subcutaneous tissue was punctured again. E) Due to the surgical technique the transplants lay on a subcutaneous tissue bridge and therefore F) the knot is separated from the transplants by subcutaneous tissue.

Fig. 2.:
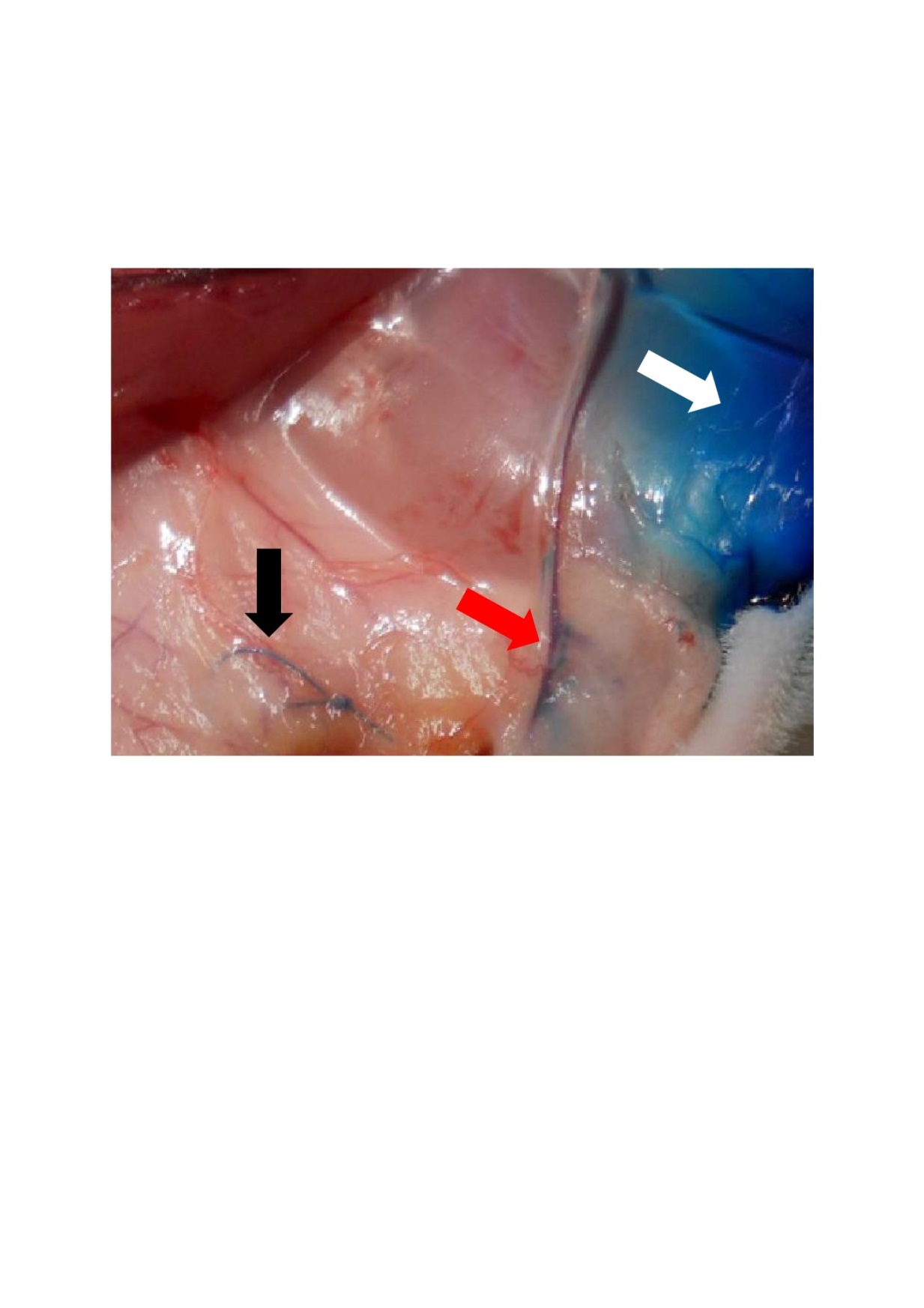


Fig. 2.: Transplantation area during sampling. The blue dye was injected in the hind leg and locally diffused in the subcutaneous tissue (white arrow) as well as it was incorporated in the afferent lymphatic collectors (red arrow). The transplant however (black arrow and marked with non-absorbable suture) was not dyed blue neither did lymphatic vessels lead to the transplant but rather grew around it (red arrow). This situation was evaluated as a transplant, which did not reconnect with the lymphatic system.
